# Evolutionary conservation of sequence motifs at sites of protein modification

**DOI:** 10.1016/j.jbc.2023.104617

**Published:** 2023-03-16

**Authors:** Shuang Li, Henrik G. Dohlman

**Affiliations:** Department of Pharmacology, University of North Carolina at Chapel Hill, Chapel Hill, North Carolina, USA

**Keywords:** AlphaFold, evolution, paralogs, proteomics, yeast

## Abstract

Gene duplications are common in biology and are likely to be an important source of functional diversification and specialization. The yeast *Saccharomyces cerevisiae* underwent a whole-genome duplication event early in evolution, and a substantial number of duplicated genes have been retained. We identified more than 3500 instances where only one of two paralogous proteins undergoes posttranslational modification despite having retained the same amino acid residue in both. We also developed a web-based search algorithm (CoSMoS.c.) that scores conservation of amino acid sequences based on 1011 wild and domesticated yeast isolates and used it to compare differentially modified pairs of paralogous proteins. We found that the most common modifications—phosphorylation, ubiquitylation, and acylation but not N-glycosylation—occur in regions of high sequence conservation. Such conservation is evident even for ubiquitylation and succinylation, where there is no established ‘consensus site’ for modification. Differences in phosphorylation were not associated with predicted secondary structure or solvent accessibility but did mirror known differences in kinase–substrate interactions. Thus, differences in posttranslational modification likely result from differences in adjoining amino acids and their interactions with modifying enzymes. By integrating data from large-scale proteomics and genomics analysis, in a system with such substantial genetic diversity, we obtained a more comprehensive understanding of the functional basis for genetic redundancies that have persisted for 100 million years.

It has long been appreciated that the yeast *Saccharomyces cerevisiae* has multiple paralogous gene pairs. However, their origins were only recognized after sequencing the complete genome, the first for any eukaryotic organism. That effort, completed in 1997, revealed a whole-genome duplication event dating back roughly 100 million years (1). Of the 6604 ORFs in this organism, there are 550 paralogous pairs (2, 3, 4), as annotated in the SGD YeastMine database (https://yeastmine.yeastgenome.org/yeastmine/begin.do). These duplicated genes are strongly enriched for components of the ribosome complex (4, 5) as well as of the glucose sensing pathway, including proteins involved in glycolysis and gluconeogenesis (6, 7). Thus, duplicated genes appear to be especially important for processes related to glucose utilization and are likely to have enabled the ability of this organism to ferment glucose to ethanol even under oxygen-rich conditions.

The prevalence of paralogous genes in *S. cerevisiae* led to broader questions about the evolutionary and selective pressures that favor their retention. Some discussions of gene paralogs have focused on their potential contributions to genetic robustness and phenotypic plasticity (8). Robustness refers to a number of different mechanisms that stabilize phenotype against genetic or environmental perturbations (*e.g.*, changes in glucose or oxygen availability). An extreme example of robustness is where one of the genes is inactivated and the remaining copy provides enough of the original function to compensate for the loss and ensure survival. In support of this model, several studies in yeast have found that about a third of duplicate gene pairs exhibit negative epistasis (9, 10, 11, 12), meaning that deleting both copies produces a significantly larger defect than that of the individual deletions. An alternative scenario is where the activity of a duplicated gene product is temporarily disabled in response to changing environmental circumstances, for example, through substrate inhibition or feedback phosphorylation. In that case, the remaining paralog might compensate for the loss by modifying its activity through transcriptional reprograming (13), changes in protein stability, or redistribution within the cell (8, 14, 15). Another feature of duplicated genes is phenotypic plasticity, which refers to the potential of new genes to evoke new phenotypes, new metabolic functions, increased biological complexity and, ultimately, the emergence of new species (16, 17) (reviewed in (18, 19, 20, 21)). These processes are not mutually exclusive; that is to say, paralogs may allow for adaptation of a given species to a broader set of environmental circumstances and at the same time help to accelerate genetic evolution.

Underlying any changes in evolution are the biochemical changes that occur within the cell. Most prominently, changes in protein function are driven by chemical modifications such as phosphorylation, glycosylation, acylation, and peptidylation (*e.g.*, ubiquitylation). A subset of these changes occurs dynamically and allows the cell to react quickly to internal and external perturbations. This was first documented in the late 1950s by Krebs and Fischer, who showed that phosphorylation at a specific serine is responsible for the interconversion of active and inactive forms of the enzyme glycogen phosphorylase (22) (reviewed in (23)). Soon after, Phillips first reported the modification of histones by acetylation (24), which disrupts DNA binding (owing to a charge reversal of modified and unmodified lysine) and alterations in chromatin structure (25). However, it would be another 25 years before the first acetylation site was mapped, in this case by epitope mapping with a monoclonal antibody specific for acetylated α-tubulin (26, 27).

Later studies revealed the importance of other posttranslational modifications. First discovered in the late 1970s, ubiquitylation entails the conjugation of a 76 amino acid protein, ubiquitin, to lysine residues in substrate proteins. This process is mediated by three distinct enzymes (E1, E2, and E3), the last of which defines substrate specificity and the timing of the modification. Once ubiquitylated, most proteins are degraded by the proteasome (28). The first use of mass spectrometry to map a protein ubiquitylation site was done for the yeast G protein Gpa1 (29). Finally, N-glycosylation is the attachment of an oligosaccharide moiety to the amide nitrogen of asparagine. This modification usually occurs on proteins destined for the cell surface, either as secreted or as integral membrane proteins. In contrast to the other modifications, N-glycosylation is considered irreversible and occurs during, rather than after, protein synthesis. Oligo-saccharyltransferases transfer a preassembled oligosaccharide from a lipid-linked donor to Asn residues within glycosylation acceptor “sequons”: Asn-X-Thr/Ser/Cys, where X is any residue other than Pro. Not all sequons are glycosylated, however, reflecting the importance of other primary and secondary structural features (30, 31). Once conjugated, these N-linked oligosaccharides undergo further processing, the products of which are specifically recognized by ER-localized lectins. Collectively, these events facilitate proper protein folding and transport to the cell surface (Reviewed in (30)).

Here, we sought to determine how paralogs in yeast differ with regard to posttranslational modifications. This was done with the expectation that chemical changes confer functional differences to otherwise structurally similar proteins and may account for the retention of duplicated gene pairs. To facilitate our analysis, we built a web-based tool that allows detailed sequence comparisons across 1012 *S. cerevisiae* strains and used this to investigate how sequence conservation near the sites of modification could account for the observed differences between paralogous protein pairs. While our analysis is limited to duplicated genes in yeast, the approach could be adapted to study other closely related protein isoforms in other organisms and thereby reveal some of the evolutionary forces responsible for their existence.

## Results

### Development of the CoSMoS.c. website

Proteins can undergo any dozens of posttranslational modifications, and these changes in chemical structure can have important biological consequences. Our initial objective was to determine if closely related proteins undergo distinct chemical modifications, and if those changes are associated with unique amino acid sequence motifs near the sites of modification. To that end, we analyzed sites of posttranslational modifications in S288C and compared the sequence of this strain with 1011 additional isolates of *S. cerevisiae* (32). These represent multiple clades from multiple geographical regions and from multiple sources in the wild, in clinical settings, in the laboratory, or used commercially in the dairy and brewing industries. In contrast to other yeasts, including commonly studied species such as *Candida albicans* and *Schizosaccharomyces pombe*, *S. cerevisiae* has a substantial number of paralogous gene pairs. In comparison to many other genomes of interest, including humans, there is much greater genetic diversity within the species *S. cerevisiae*. Most importantly, and in contrast with previous studies, we restricted our analysis to modified and unmodified pairs of paralogous proteins. This represents a very powerful test for the hypothesis because paralogs have a shared evolutionary history and are expected to have similar secondary structures. Moreover, the use of within-species polymorphism data is much less susceptible to the alignment errors that often occur with longer evolutionary comparisons.

**Figure 1:**
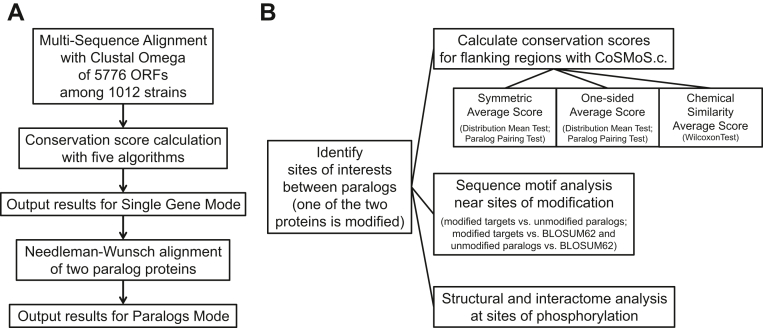
**Workflow of analysis.** Analysis outline for (*A*) CoSMoS.c. and (*B*) statistical analysis. CoSMoS.c., Conserved Sequence Motifs in *Saccharomyces cerevisiae*.

**Figure 2:**
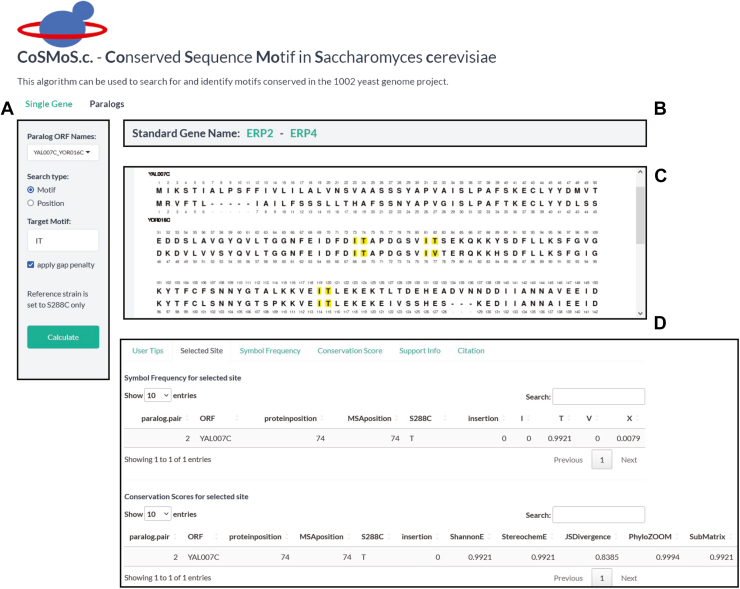
**The landing page for the CoSMoS.c. website contains input and output display panels.** There are two search options: Single Gene and Paralogs. Shown is a paralog search. *A*, the input panel contains options for search type, ORF name, reference strain name, and whether or not to apply a gap penalty. The reference strain is used for the PhyloZOOM algorithm. The gap penalty, when applied, decreases the score if there are nonstandard amino acids at the target site. The search type can be either by motif or by position within the sequence. The output panel contains three sections (*top*, *middle*, *bottom*). *B*, the *top section* shows the standard gene names and contains a hyperlink to the corresponding pages of the SGD website. *C*, the *middle section* shows paralog sequences aligned using Needleman-Wunsch with the amino acids numbered. A position or motif that matches the input is indicated by highlighting. The highlighted region is interactive; that is to say, clicking on a highlighted amino acid will display the Frequency Table and Conservation Score table under the “Selected Site” tab (*D*). The *bottom panel* contains five additional tabs. “User Tips” provides brief instructions on how to input data and what to expect in terms of output. “Support Info” provides details on how to interpret the output statistics and a hyperlink to a detailed user manual. “Citation” tab shows how to reference the CoSMoS.c. website. “Symbol Frequency” and “Conservation Score” provide statistics for all matched sites, as detailed in Figs. S1 and S2, respectively. CoSMoS.c., Conserved Sequence Motifs in *Saccharomyces cerevisiae*.

To begin our analysis, we first performed multisequence alignment (using Clustal Omega 1.2.4) for 5776 ORFs shared among the 1012 strains (Fig. 1*A*, see Experimental procedures) (32). We then used these alignments to identify and analyze specific sites of interest (described below), which were the foundation for subsequent conservation score calculations. We also created an interactive website, CoSMoS.c. (Conserved Sequence Motifs in *Saccharomyces cerevisiae*, https://shiny-server-dept-yeast-cosmos.apps.cloudapps.unc.edu/) that allows users to identify, either by sequence motif or by position within the sequence, and score the conservation of aligned regions of any protein or paralog pair (Figs. 2, S1, and S2). For Single Gene mode, the conservation score of any motif or position can be calculated. For Paralogs mode, the two proteins are aligned with the Needleman–Wunsch global alignment and displayed on the sequence map (Fig. 2*C*), with conservation score calculated for desired motifs or positions. To address multiple potential applications, we included five widely used algorithms to calculate conservation scores (see ‘Conservation score calculation’ in Experimental procedures for details): Shannon Entropy, which reports the average level of uncertainty (or “information” or “surprise”) inherent in the possible outcomes of the variable and thereby quantifies amino acid diversity at a given position (33); Stereochemically Sensitive Entropy, which is based on Shannon Entropy but groups amino acids into nine categories based on similarities in their physiochemical properties (34); PhyloZOOM, which weights evolutionary relatedness on top of chemical identity (32, 35); Jensen-Shannon Divergency (JSD), which compares amino acid frequencies with the background distribution, assuming no evolutionary constraint, and thereby emphasizes selection pressure rather than chemical similarity (36); and Karlin Substitution Matrix, which quantifies the likeliness of observed substitutions, rather than the chemical or biological properties of a given amino acid (37). Thus, each algorithm considers a different aspect of amino acid sequence, which when used together provides a more comprehensive representation of protein conservation.

### Statistical analysis using CoSMoS.c.

Soon after the completion of the yeast genome sequencing project, many large-scale investigations of protein modifications were conducted using mass spectrometry. One potential, and particularly powerful, use of CoSMoS.c. is to determine whether specific modifications occur in regions of high sequence conservation. To that end, we aligned the sequences of paralogous protein pairs and selected those amino acid sites that are the same in both paralogs, but where only one of the two sites is modified (Fig. 1*B*). We then used CoSMoS.c. to calculate the conservation scores for those sites of interest and their flanking regions. Our underlying hypothesis was that if there are sequences that favor a given modification, such sequences are likely to flank the site of interest, and those sequences are likely to be conserved.

To maximize statistical power, we focused on the five most common modifications in *S. cerevisiae*: phosphorylation, with 38,684 occurrences present in 4120 ORFs (62.39%), ubiquitylation, with 5299 occurrences in 1872 ORFs (28.35%), monoacetylation, with 968 occurrences in 333 ORFs (5.04%), N-glycosylation, with 587 occurrences in 239 ORFs (3.62%), and succinylation, with 577 occurrences in 356 ORFs (5.39%) (Table 1 and Dataset S1), as annotated for strain S288C in the SGD database (https://yeastmine.yeastgenome.org/yeastmine/begin.do). Phosphorylation of serine, threonine, and tyrosine has been documented 30,029, 7723, and 932 times, respectively (Table 1). The abundance of phosphorylated tyrosines was unexpected given that in yeast, there are no dedicated tyrosine kinases and only a small number of dual-specificity kinases. We did not include rare noncanonical events, including phosphorylation of other amino acids or events annotated as “dephosphorylation” or “autophosphorylation”. All of these modifications can affect protein activity, location, or protein–protein interactions.

**Table 1:** Most common modifications in the complete proteome (all proteins) of *Saccharomyces cerevisiae* were obtained from annotated lists in the SGD database and assigned to 550 paralogs (paralog proteins)

Modification type	Amino acid	All proteins	Paralog proteins			
		Total	Needle conserved		Total	Conserved
			FALSE	TRUE		
Phosphorylation	S	30,029	4997	3290	8287	39.70%
Phosphorylation	T	7723	1329	797	2126	37.49%
Phosphorylation	Y	932	147	109	256	42.58%
Phosphorylation	S/T/Y	38,684	6473	4196	10,669	39.33%
Ubiquitylation	K	5299	632	1160	1792	64.73%
(Mono)acetylation	K	968	49	306	355	86.20%
N-glycosylation	N	587	48	91	139	65.47%
Succinylation	K	577	77	471	548	85.95%

Shown are instances where a modified amino acid is conserved (TRUE) or not conserved (FALSE) with the corresponding site in its paralog, whether or not that site is modified in the paralog. Corresponding sites were identified by aligning paralogous protein sequences using the Needleman algorithm (needle conserved). Details of the frequency of modifications in protein paralog pairs are provided in Dataset S1.

We next identified pairs of sites in which one of the two proteins is known to be modified and the amino acid residue is identical in both (“site of interest”) (Fig. 1*B*). These sites were identified by aligning sequences using the Needleman algorithm (38). We did not consider substitutions of any kind even if they are potentially modified (*e.g.*, serine for threonine). By restricting our analysis in this way, we make no assumptions about amino acid substrate preferences for modifying enzymes. Then, we sought to determine whether the modifications occur within a region of high amino acid conservation or conserved sequence motif. To that end, we examined the flanking region of each site of interest using three methods of analysis and five different scoring algorithms within CoSMoS.c. (see Experimental procedures). We are not inferring any causal relationship between a particular modification and the sequence context of that modification, particularly since different substrates may undergo the same chemical reaction but are carried out by different modifying enzymes. Further experimentation is needed to determine if a particular sequence helps to direct a specific enzyme to the site of modification or if a given modification favors retention of sequences that are functionally compatible with a given posttranslational modification.

**Figure 3:**
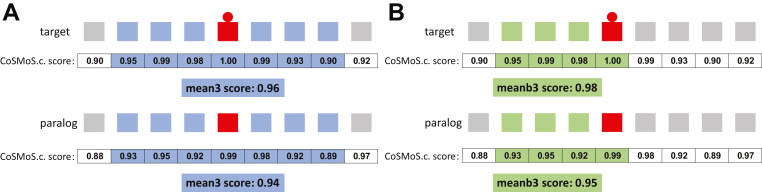
**Schematic illustrating symmetric average score and one-sided average score.***A*, symmetric average calculation of mean3 score for a given modified target protein and its unmodified paralog. *B*, one-sided average calculation of meanb3 score for a given modified target protein and its unmodified paralog. *Red circle* and *squares* show the modified amino acid in the target protein. *Red square* shows the conserved amino acid in the paralog protein without modification. Amino acids and corresponding conservation scores used for mean3 or meanb3 score calculation are shaded in *blue* or *green*.

**Figure 4:**
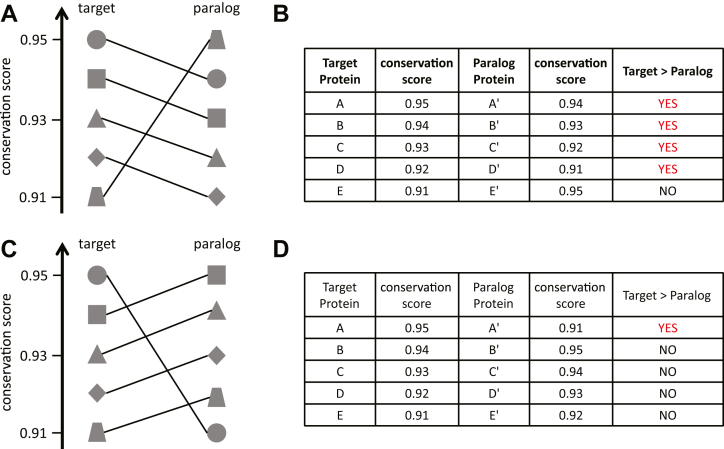
**Schematic illustrating how pairing structure can advantage or disadvantage target proteins.** In all panels, hypothetical target and paralog proteins have the same conservation scores, but the pairing structure is such that target proteins have higher scores in four out of five instances (*A* and *B*) (advantage) or target proteins have higher scores in only one out of five instances (*C* and *D*) (disadvantage).

The first method, which we call symmetric average score, considers the conservation scores for sets of amino acids upstream and downstream of the site of interest. That is to say, we obtained mean scores for sets of amino acids that include, and bracket on both sides, the sites of interest. These scores are represented as mean1 (three amino acids), mean2 (five amino acids), mean3 (seven amino acids), and mean4 (nine amino acids) (Fig. 3*A*). To determine whether the modified target sites have a higher context sequence conservation than that of the unmodified paralogs, we first separated the scores into five groups, one for each modification type. We then performed two statistical tests: the distribution mean test, which determines whether target protein conservation score distribution (that is, conservation scores for all modified target proteins) is significantly larger than the distribution of the unmodified paralogs, and the paralog pairing test, which tests whether the pairing structure confers an advantage for the target proteins. Figure 4 presents two possible pairing structures (panels A and C) and how these can advantage (panels A and B) or disadvantage (panels C and D) target proteins. We also list how these two tests are expected to perform under all possible scenarios with regard to the relationships between modified target proteins and unmodified paralogs (see Experimental procedures).

The second method we call one-sided average score. In contrast to symmetric average score, which simultaneously considers sequences on both sides, this alternative method considers up to four amino acids either upstream or downstream (but not both) of the site of interest (Fig. 3*B*). As with the first method, we compared the score for each paralog pair, separated the scores into the five modification types, and performed the same statistical tests on the score distribution.

The third method, which we call chemical similarity average score, calculates the mean conservation score based on chemical classifications assigned to residues immediately adjacent to a site of interest. That is to say, we obtained a mean score for amino acids, comprised of the site of interest and the residue immediately before (“meanb1”) or immediately after (“meana1”). We then placed each of these amino acids into five separate bins based on the chemical classification of the adjoining residue, as follows: aliphatic (G,A,V,L,I,M,P), aromatic (F,Y,W), polar uncharged (S,T,C,N,Q), acidic (E,D), and basic (K,R,H). We then compared the modified target proteins and their unmodified paralogs, by calculating the average of meanb1 and of meana1, for each classification. Because the residues of a given target and its paralog could fall into two different chemical classifications, the paralog pairing structure cannot be maintained in this analysis. Therefore, we only applied the Mann-Whitney-Wilcoxon test to compare the means for the target and paralog distribution of a certain chemical classification. In this way, we determined whether there was a significant difference in conservation scores between modified target proteins and unmodified paralogs for each chemical group. Thus, we could determine if the occurrence of a specific modification depends on the physiochemical properties of nearby amino acids.

### Analysis of sequence conservation near sites of posttranslational modification

Having established our analytical approach, we next sought to apply it to specific posttranslational modifications. To that end, we focused on five major modifications, representing those with more than 500 documented occurrences in the proteome of *S. cerevisiae*, as annotated in the SGD YeastMine database (https://yeastmine.yeastgenome.org/yeastmine/begin.do): phosphorylation, N-glycosylation, monoacetylation, succinylation, and ubiquitylation. More specifically, we sought to identify patterns of sequence conservation near each modification site and to determine how any such sequence motifs differ depending on the type of modification.

**Figure 5:**
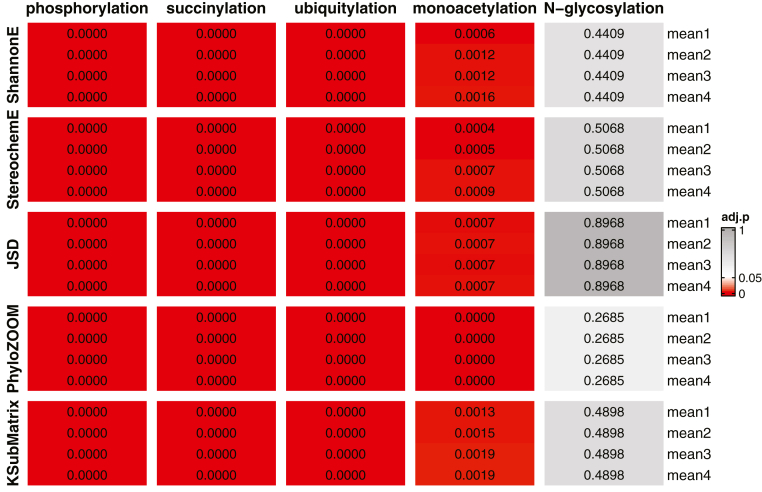
**Results of distribution mean test for symmetric average score.** Displayed is a heatmap of adjusted *p* values for all five algorithms with different flanking range (mean1 to mean4, rows) for each modification type (columns). *Gray*, *p* > 0.05; *white*, *p* = 0.05; *red*, *p* < 0.05. Precise values are provided in Dataset S2.

**Figure 6:**
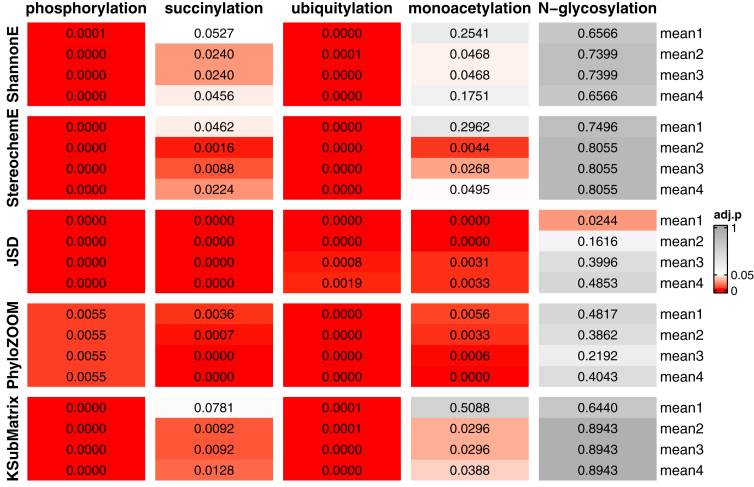
**Results of paralog pairing test for symmetric average score.** Displayed is a heatmap of adjusted *p* values for all five algorithms with different flanking range (mean1 to mean4, rows) for each modification type (columns). *Gray*, *p* > 0.05; *white*, *p* = 0.05; *red*, *p* < 0.05. Precise values are provided in Dataset S3.

We first applied the Mann-Whitney-Wilcoxon (Distribution Mean) and Monte Carlo Simulation (Paralog Pairing) tests to the symmetric average score. Figures 5 and 6 show the adjusted *p* values (Benjamini-Hochberg, same method used for all adjusted *p* values) for each of the five modifications, one per column, as applied to all four sequence lengths, one per row. For the distribution mean test, we found significant differences (adjusted *p* < 0.05) in conservation scores comparing modified target proteins and unmodified paralog proteins, for all modifications except N-glycosylation (Fig. 5 and Dataset S2). We also found significant differences for all sequence lengths, from one to four amino acids, flanking the site of interest. This suggests that there is a functional relationship between these modifications and their adjoining sequences. Once again, we are not inferring any mechanistic relationship between any modification and the sequence context of that modification.

We then applied the paralog pairing test to each modification type using all five algorithms (Fig. 6 and Dataset S3). Once again, conservation scores for phosphorylation and ubiquitylation were significantly different for all four sequence lengths and for all five algorithms. Succinylation had borderline adjusted *p* values for mean1 scores obtained using Shannon Entropy and Karlin Substitution Matrix. While monoacetylation had significant adjusted *p* values for mean2, mean3, and mean4, the adjusted *p* values were significant for just two of five mean1 scores (JSD and PhyloZOOM). JSD reports how much we expect the amino acid sequence to change assuming no evolutionary constraint. The JSD value was greater for modified targets than unmodified paralogs. Therefore, the significant adjusted *p* value suggests that selection pressure is greater near modified sites than unmodified sites. In contrast to the other algorithms, PhyloZOOM penalizes mutations according to phylogenetic distance. We found that for mean1 of monoacetylation, the modified target proteins are more likely to have a PhyloZOOM score that is equal to or greater than that of unmodified paralogs. Accordingly, the sequences of unmodified paralogs exhibit substitutions in strains closely related to S288C, while modified proteins harbor substitutions in distantly related strains. In summary, JSD indicates that there is selection pressure to ensure the conservation of sequences flanking sites of modification, and these forces are relaxed for corresponding sites in unmodified paralogs. PhyloZOOM indicates that the selection pressure for modified regions is greater in strains most closely related to S288C. Such differences are less likely to be detected when using other algorithms such as Shannon Entropy.

Based on these data, we infer that the pairing structure helps to amplify differences between target proteins and their paralogs, and these differences are evident for phosphorylation, ubiquitylation, succinylation, and monoacetylation, but not for N-glycosylation. We obtained similar results using the one-sided average score, which considers up to four amino acids either upstream or downstream of the site of interest, but not both. For both the distribution mean test and the paralog pairing test, adjusted *p* values were significant for segments upstream and downstream of the sites of phosphorylation, succinylation, ubiquitylation, and monoacetylation, but not N-glycosylation (Figs. S3 and S4; Datasets S4 and S5).

**Figure 7:**
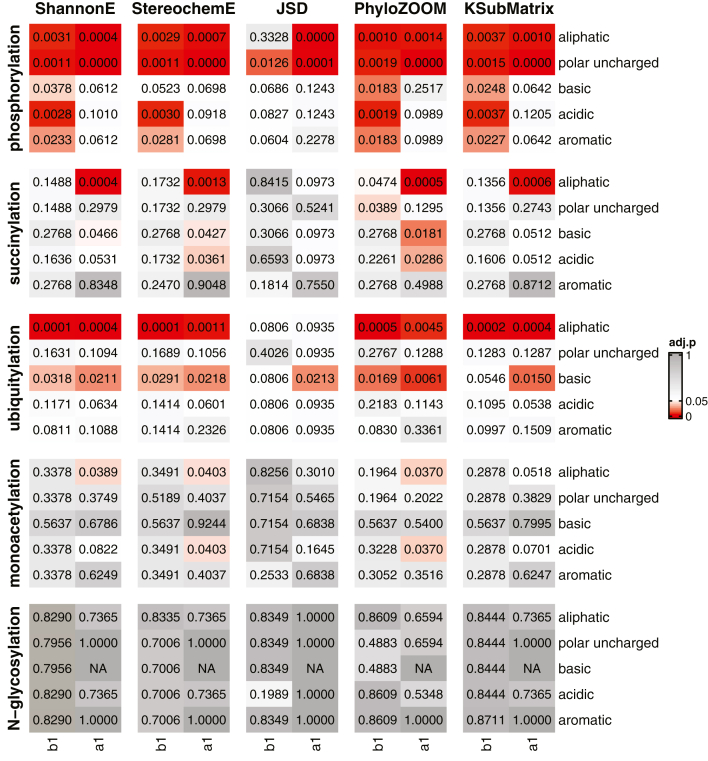
**Results for chemical similarity average score.** Displayed are adjusted *p* values for all five algorithms (column pairs) and all five modifications (rows). For each modification type, amino acid categories immediately adjacent to the site of interest (first column, upstream-b1; second column, downstream-a1) are plotted separately. *Gray*, *p* > 0.05; *white*, *p* = 0.05; *red*, *p* < 0.05. Precise values are provided in Dataset S6.

Finally, we determined the chemical similarity average score, which calculates the mean conservation score based on chemical classifications assigned to amino acid residues. We used the Mann-Whitney-Wilcoxon test to compare modified target proteins and unmodified paralogs for each chemical classification. Once again, we observed significant differences for all modifications except N-glycosylation (Fig. 7 and Dataset S6). In particular, we found high conservation of aliphatic residues flanking sites of phosphorylation and ubiquitylation and immediately after sites of succinylation. In addition, we found high conservation of basic residues flanking sites of ubiquitylation and of polar uncharged residues flanking sites of phosphorylation. Notably, we observed a significant difference for all five amino acid classifications upstream of the sites of phosphorylation. This result indicates the importance of conservation at this position, one that is independent of the chemical properties of the amino acids at that position. One possibility is that protein kinases share the ability to recognize amino acids immediately before the site of modification, but that dependence may differ for different kinases. More broadly, we conclude that a subset of amino acids is conserved near sites of protein phosphorylation, succinylation, and ubiquitylation. This is true even when there is no obvious ‘consensus site’ for a given modification. This pattern of conservation could indicate regions that are functionally important and happen to undergo posttranslational modification. Alternatively, flanking sequences could favor recognition by the enzymes that confer these modifications or disfavor recognition by the enzymes that remove them. Further studies are needed to establish a cause-effect relationship between each modification, modifying enzymes, and the sequence context of the various modifications.

We then reanalyzed our data to account for possible effects of protein abundance, which in cross-species comparisons was observed to negatively correlate with evolutionary rate and positively correlate with modification detection by mass spectrometry (39). Accordingly, we restricted our in-species analysis to a subset of 270 paralog pairs that have similar (<2-fold difference) median abundances. After applying this filter, we were left with >100 instances each of phosphorylation, ubiquitylation, and succinylation, where the target and paralog have the same amino acid, but only the target is modified. Even with this restricted dataset, we obtained similar results for all three types of analysis (Dataset S7). We also considered the potential effect of false positives and false negatives among the reported modification sites. False positives can result from ambiguous assignments, as might arise through misidentification of modified sites within peptides that contain multiple potential sites of modification. False negatives can result from difficulties in detecting modifications in poorly expressed proteins (39) or an overly strict reliance on high confidence sites. We then further restricted the data to only include modifications identified in multiple studies. After applying this additional filter, we were left with >100 instances of phosphorylation. Once again, we obtained similar results for symmetric average score and one-sided average score analysis, but not for chemical similarity average score, which is further restricted by splitting the data into five chemical categories (Dataset S8).

### Analysis of sequence motifs near sites of posttranslational modification

We next examined specific features of amino acids flanking the sites of modification and paid particular attention to motifs unique to modified target proteins (Table 2). In this analysis, we focused on phosphorylation, since it is well established that many protein kinases recognize specific amino acid residues at positions near the site of phosphorylation (40, 41) (https://services.healthtech.dtu.dk/service.php?NetPhos-3.1); (https://scansite4.mit.edu/#home). To that end, we used the Chi-square test, to determine whether the amino acid distribution for a specific site differs between modified targets and unmodified paralogs, and performed post hoc analysis to determine which amino acid underlies this difference (see Experimental procedures) (42, 43, 44). Not surprisingly, most of the statistically significant differences were observed for serine phosphorylation, which is more common than phosphorylation of threonine or tyrosine. In this instance, we detected significant or marginally significant enrichment of arginine at position b3, glycine at position b1, and proline at position a1. Conversely, certain amino acids were disfavored at positions b3 (asparagine) and a1 (serine, threonine, tyrosine, and lysine). There is good agreement with the sequence features identified in our analysis and that of a recent analysis using unbiased combinatorial peptide library screening methods to determine substrate specificity determinants for 303 purified serine-threonine kinases in humans (45). In that analysis, nearly half of the kinases could be assigned to sequence motifs enriched either in basic residues at the b3 and b2 positions or a proline at the a1 position.

**Table 2:** Analysis of sequence motifs near sites of posttranslational modification

Modification	Relative position	Amino acid	Relative abundance	Adjusted *p* value
**Phosphorylated S**	**b3**	**R**	**Target > Paralog**	**<0.0001**
Phosphorylated S	b3	N	Target < Paralog	0.0007
**Phosphorylated S**	**b1**	**G**	**Target > Paralog**	**0.0641**
Phosphorylated S	a1	S	Target < Paralog	0.0442
Phosphorylated S	a1	K	Target < Paralog	0.0262
Phosphorylated S	a1	T	Target < Paralog	0.0262
Phosphorylated S	a1	Y	Target < Paralog	0.0949
**Phosphorylated S**	**a1**	**P**	**Target > Paralog**	**<0.0001**
**Phosphorylated T**	**a1**	**P**	**Target > Paralog**	**0.0675**

Shown are flanking amino acids before (b3, b1) and after (a1) favored (bold) or disfavored in modified target proteins, as compared to their unmodified paralog.

For the other types of modifications, we did not observe any significant differences between target and paralog (Table 2). However, we did observe some differences in amino acid abundances, compared to that of BLOSUM62, which is denoted as the background amino acid frequencies approximating those with no selection pressure (see Experimental procedures) (46). Therefore, significant difference from BLOSUM62 distributions can be viewed as being constrained by evolution or having functional importance. For sites of monoacetylation, positively charged amino acids (lysine and arginine) were rarely present at the preceding (b1) position (Fig. S5*A*). These findings agree with, and substantially expand on, prior work showing that acetylation occurs in regions enriched in Lys, Ser, Thr, Gly, and Ala (47). For succinylation, the subsequent (a1) site was enriched for lysine, aspartic acid, and cysteine (Fig. S5*B*). As with monoacetylation, the b1 position lacked arginine (Fig. S5*C*). N-glycosylation, like phosphorylation, occurred in regions with an abundance of serines and threonines; these residues were almost exclusively found at the a2 position, in accordance with the known consensus site for N-glycosylation, as noted above (Fig. S5*D*).

### Analysis of structural and interactome differences at sites of phosphorylation

Above, we identify several thousand instances where one of two paralogous proteins undergoes a unique posttranslational modification and describe an algorithm that scores sequence conservation across 1000+ isolates of the same species. We then considered two possible mechanisms by which the differences in modification might arise. First, we considered secondary structure. Modifications such as phosphorylation often occur in regions that lack obvious secondary structure; previous estimates indicate that 75% or more of phosphorylation sites, 45% of ubiquitylation sites, and 40% of acetylation sites lie within disordered regions of proteins, particularly proteins expressed in the cytoplasm (48, 49, 50, 51). For example, in a recent analysis of over 900 yeast proteins with experimentally determined structures, more than two thirds (5943 of 8708) of known phosphorylation sites mapped to regions that are disordered (52). The lack of structure is thought to provide increased accessibility to modifying enzymes. Second, we considered the relationship between experimentally determined phosphorylation events and documented interactions with protein kinases. Any such differences could account for the differences in phosphorylation reported here.

In order to determine the role of secondary structure, we mapped phosphorylation sites onto predicted protein three-dimensional structures available through AlphaFold (53, 54). This algorithm provides highly accurate structure prediction by incorporating novel neural network architectures and training procedures based on the evolutionary, physical, and geometric constraints of protein structures. We downloaded all available AlphaFold protein structure predictions for *S. cerevisiae* and used STRIDE to assign residue solvent accessible area and secondary structure for each amino acid (55). We then matched secondary structure predictions to each of the modified target sites and the corresponding unmodified paralog pairs (Dataset S9). As shown in Fig. S6, the most frequently observed structures for the target-paralog pairs are Coil-Coil (1363), AlphaHelix-AlphaHelix (712), Turn-Turn (400), and Strand-Strand (243), accounting for more than 80% of the total (3343). Therefore, there are no substantial differences in secondary structure type comparing targets and paralogs. We then examined each paralog pair for differences in secondary structure length or residue solvent accessible area. For these four most common secondary structure pairings, the distribution of the difference between target *versus* paralog for secondary structure length (Fig. S6*A*) and residue solvent accessible area (Fig. S6*B*) all centered near 0, with sides that were not significantly skewed in either direction. We conclude that secondary structure and residue solvent accessible area are unlikely to account for the functional differences between target and paralog.

**Figure 8:**
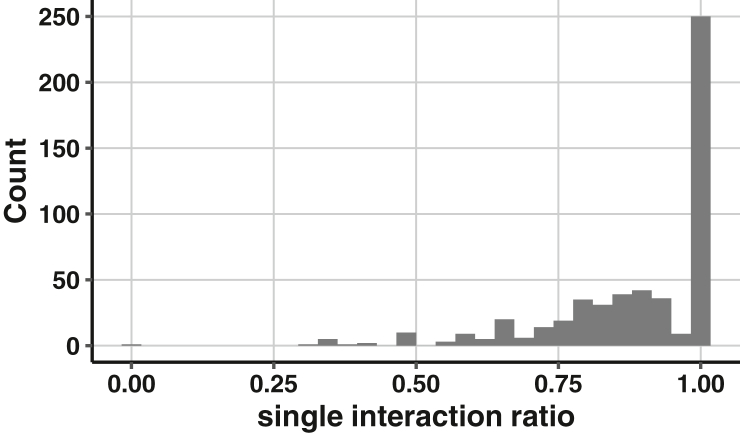
**Histogram of single interaction ratio for all paralog pairs.** Single interaction ratio, the number of kinases that interact with one but not both paralogs divided by the sum of the number of kinases that interact with one or both paralogs.

We next considered the possibility that the paralogous proteins have distinct binding partners and these differential interactions could account for the different modifications observed. To that end, we interrogated the Yeast KID–kinase interaction database (http://www.moseslab.csb.utoronto.ca/KID/index.php) (56), representing a total of 31,155 documented interactions between protein kinases and potential substrates. Of these, 7142 are interactions of protein kinases with paralogs. Because of limitations of the data source, we cannot assign a specific kinase to any particular site of modification. Therefore, we performed our analysis with all 550 paralogous protein pairs instead of individual sites in the modified target *versus* unmodified paralog. For each of the paralog pairs, we counted the number of kinases that interact with both proteins (double interaction) and the number that interact with only one of the two proteins (single interaction). We then calculated the single interaction ratio as the number of kinases with the single interaction divided by the sum of kinases with either single or double interactions. From this analysis, we identified 12 paralogous pairs that do not interact with any kinase. Out of the remaining 538 paralogous pairs, 250 had a single interaction ratio of 1 (Fig. 8 and Dataset S10), meaning that these kinase interactions are exclusively with one of the two paralogous proteins, but not both. In comparison, if kinases interacted equally with both paralogous proteins, we would have a single interaction ratio of 0.

Finally, in an initial effort to match sites of phosphorylation with protein kinases, we used the position-weight matrices (PWMs) developed by Mok *et al.* (57, 58). That analysis determined phosphorylation site selectivity for 61 of the 122 kinases in *S. cerevisiae* and proposed empirically derived PWMs that enable the assignment of candidate protein kinases to known sites of phosphorylation (57, 58). We applied the PWMs to our dataset, which contains sites where one of the two proteins is known to be phosphorylated and the amino acid residue is the same in both. From this dataset, we kept 190 paralogous pairs where each protein contains at least one such phosphorylation site, so that both proteins would have kinase interactions to be compared. Using the PWMs from (58), we assigned the kinase that most likely corresponds to each phosphorylation site, as implemented in (57). Out of the 190 paralogous pairs, 130 interacted with a completely different set of protein kinases (Dataset S11). That is to say, for 130 pairs of paralogs, one but not the other interacted with a given kinase. Together, these results indicate that most kinases regulate one or the other of the protein paralogs. They suggest further that differential modifications reported here may be the result of differential interactions with modifying enzymes.

## Discussion

Posttranslational modifications are critical functional elements within proteins and are therefore expected to be conserved in evolution. Here, we have identified several thousand instances where, despite a shared ancestry, only one of two paralogous proteins undergoes a specific posttranslational modification. We also developed a custom algorithm that quantifies sequence conservation, in an automated fashion, across 1012 unique strain isolates. By comparing adjoining sequences in multiple isolates of the same species, we determined that sequence conservation near sites of modification is greater than at sites that are not modified. In addition, many of the modifications were associated with conserved sequence elements nearby. We postulate that these differences in sequence conservation are partly responsible for differences in enzyme recognition as well as posttranslational modifications, that differences in posttranslational modifications allow duplicated proteins to be differentially regulated, and these differences may account for their retention after 100 million years of evolution.

Our analysis is clearly distinct from, and complementary to, earlier investigations of posttranslational modifications in yeasts. Previous analysis showed that duplicated proteins in *S. cerevisiae* are more likely to be phosphorylated and to have a greater number of phosphorylation sites, than nonduplicated proteins (59). The difference persisted when controlling for differences in protein abundance, coverage, essentiality, positioning within protein interaction networks, and assembly into multiprotein complexes (59). When compared with a yeast species that diverged before the whole-genome duplication event, it appears that the majority of phosphorylation sites in paralogs have either been lost or gained, with a strong bias toward losses (57). Subsequent *cross-species* comparisons noted a high degree of sequence conservation near sites of phosphorylation and other types of modification in yeasts (50, 60, 61, 62, 63, 64, 65, 66). The relationship was strongest for phosphosites with known function (50, 51, 62). A focused study of 249 unique high-confidence phosphorylation sites, targeted by seven protein kinases in *S. cerevisiae*, confirmed that regions flanking sites of phosphorylation are significantly constrained, in comparison with other closely related yeast species (62). A similar relationship exists for sites phosphorylated by the cyclin-dependent protein kinase Cdk1 (67) and was the basis for predicting novel sites of phosphorylation by the cAMP-dependent protein kinase (68). Our analysis builds on these foundational studies, by considering new and substantially larger proteomics datasets, multiple additional types of posttranslational modifications, new and sophisticated models of protein structure, large-scale kinase interactome data, and *in-species* sequence conservation data, with particular emphasis on duplicated proteins. The contrast between *cross-species* and *in-species* analysis is particularly important because differences in evolutionary pressure may evoke differences in structural and functional conservation.

We propose that in-species comparisons of paralogs will prove to be more reliable than cross-species comparisons of orthologous proteins or in-species comparisons of nonhomologous proteins. Comparisons of paralogs are powerful because they are likely to have similar structures and related functions, due to their shared evolutionary origin (57, 59, 69). Comparison within a single species is powerful because it allows us to avoid important nonbiological sources of uncertainty, such as potential alignment errors and unknown structural or functional differences. This is supported by a small number of prior studies, which compared four sets of paralogous proteins in yeast—Rck1 v. Rck2, Fkh1 v. Fkh2, Ace2 v. Swi5 (69), and Boi1 v. Boi2 (59)–and concluded that divergence in short linear motifs is likely responsible for differences in phosphorylation. A similar conclusion emerged from a comparison of predicted sites of phosphorylation in mammalian p53, p63, and p73 (70).

Our analysis of differentially modified pairs of paralogous proteins revealed that the most common modifications—phosphorylation, ubiquitylation, and acylation but not N-glycosylation—occur within regions of high sequence conservation. Further studies will benefit from the availability of our search algorithm CoSMoS.c.. For example, when studying a particular protein kinase, CoSMoS.c. can be used to identify specific motifs near potentially modified serines, threonines, and tyrosines (Table 2). When studying a particular substrate of ubiquitylation, CoSMoS.c. can be used to prioritize conserved *versus* nonconserved sequences flanking potentially modified lysines. For rare modifications, CoSMoS.c. can also be used to locate highly conserved regions as the starting points for finding new sequence motifs. We note that conservation near sites of modification is likely correlated with overall conservation of the paralogs and, further, that differences in posttranslational modification are likely to be particularly important for paralogs that are the most closely related in sequence. Moreover, there may be some bias towards well-characterized kinase-substrate pairings, although we have attempted to address the concern by acquiring the most up to date datasets arising from a combination of low- and high-throughput methods. Finally, it would be of interest to determine how posttranslational modifications influence, and are influenced by, differences in protein subcellular localization. Thus, by comparing unique modifications in closely related gene products and across closely related strain isolates, we can prioritize mechanistic investigations of modifications that are likely to have functional importance, to identify recognition motifs for specific modifying enzymes, and to better predict new enzyme-substrate relationships.

As noted above, a particular advantage of working with *S. cerevisiae* is the existence of several hundred paralogous gene pairs, most with well-annotated functions. However, their persistence over millions of years has remained a puzzle. Duplicated genes are inherently unstable, and one or the other copy is likely to accumulate mutations and become a pseudogene or be eliminated entirely (71, 72, 73). Despite these countervailing forces, a substantial number of paralogs has been retained, presumably because these duplication events confer some fitness advantage to the organism. One potential benefit of gene duplication is to increase protein expression and metabolic flux (74, 75). In support of this dosage amplification model, many paralogous gene pairs exhibit substantial functional redundancy, as indicated by the high frequency of shared protein interaction partners (76, 77). On the other hand, it has been difficult to identify any growth or metabolic phenotypes following deletion of individual paralogs (9, 10, 11, 12, 78). Even a combined deletion of 13 single paralogs, each involved in the conversion of glucose to ethanol, revealed no defect with respect to gene expression, the formation of glycolytic products, or growth in a variety of conditions (6). Similarly, a combined deletion of 24 single paralogs associated with 40S ribosomes led to only mild loss of translation activity and cellular fitness (79). One possibility is that differences in fitness exist but are evident only under highly specialized (nonlaboratory) circumstances. Another possibility is that deletion of a given paralog leads to a compensating change in the remaining paralog, through alterations in gene transcription (13, 17), protein half-life (80), or protein abundance (81). Given this complication, and in contrast to most prior studies which examined the functional consequences of individual gene deletions, it is noteworthy that we focused our analysis on events that occur in an otherwise WT background.

Another potential benefit of gene duplication is the acquisition of new functions or neofunctionalization. In this scenario, one copy might retain the original function, while the second is free to acquire novel functions that are genetically favored (3). In support of the model, an analysis of published fitness data revealed that combined deletion of paralogous pairs in *S. cerevisiae* exhibit a stronger defect than that of the corresponding singletons in *S. pombe*, which did not undergo the same whole-genome duplication event (82). The expanded functionality of duplicates appears to include a larger number of protein-binding partners and increased presence within multiprotein complexes (83, 84, 85, 86, 87, 88). We propose that many of the 3500+ unique modifications—those occurring in one of two duplicated proteins—are the consequence of differential binding to regulatory enzymes, as shown here for protein kinases (Fig. 8).

A third potential outcome of gene duplication is subfunctionalization, where the function of a single ancestral protein becomes distributed among two descendant proteins. It has been argued, based on analysis of genetic and protein interactions as well as directed evolution studies, that subfunctionalization is associated with whole-genome duplications, while neofunctionalization is most characteristic of small-scale duplications (12, 83, 87). By this mechanism, duplicates can be maintained in the genome by acquiring reciprocal loss-of-function mutations, such that both duplicates become necessary to perform the combined functions of a common ancestor. These functions are likely to include distinct regulation by phosphorylation (57, 89) and changes in catalytic activity (90) or subcellular localization (91, 92). However, nearly all prior studies of subfunctionalization have focused on differences in transcriptional regulation (7, 76, 78, 93, 94, 95, 96, 97, 98). In contrast, we focused our analysis on changes that occur later, through posttranslational modifications of the encoded proteins.

By working with yeast, we circumvent many of the challenges associated with more complex biological systems. Nevertheless, our investigations in yeast could help to explain the prevalence of seemingly redundant protein isoforms in humans. Whereas some paralogous pairs have partitioned their functions through changes in protein sequence alone, others are likely to have acquired new roles through posttranslational modifications. These observations provide a possible explanation for the retention of functionally similar proteins throughout evolutionary history.

## Experimental procedures

### Multisequence alignment

Multisequence alignments were performed using the reference strain S288C as well as 1011 other strains provided by the “1002 Yeast Genome” website (http://1002genomes.u-strasbg.fr/files/) (32). Gene sequences comprising 6015 nonredundant ORFs were downloaded from the allReferenceGenesWithSNPsAndIndelsInferred.tar.gz file. To simplify our analysis, the 239 intron-containing genes were not considered, leaving 5776 ORFs. The S288C and “1002” datasets were combined for a total of 1012 strain sequences. These gene sequences were translated into protein sequences with the ‘translate’ function in seqinr package in R (https://cran.r-project.org/web/packages/seqinr/index.html). The translated protein sequences were used as input for Clusto Omega (version 1.2.4), with the arguments ‘--seqtype=Protein --infmt=fasta --outfmt=fasta --guidetree-out=user_defined_routes --use-kimura --iter=2 --force’, to produce multisequence alignments. The output was 5776 files, one for each protein, each containing an alignment for the 1012 strains.

For each site of modification, the Needleman–Wunsch global alignment was performed to identify corresponding regions in each paralogous protein pair (38). The function 'pairwiseAlignment' in the R package 'Biostrings (v3.14)' and the following arguments were used: type = 'global', substitutionMatrix = 'BLOSUM62', gapOpening = 10, gapExtension = 0.5, scoreOnly = F.

### Conservation score calculation

To calculate conservation scores, we applied five commonly used algorithms. Each algorithm considers a different aspect of amino acid sequence, which when used together provides a more comprehensive representation of protein conservation. The code used to calculate the scores is available for download (https://github.com/Shuang-Plum/YeastMotifConserv).

In CoSMoS.c., Shannon Entropy was calculated as described previously (33) and was defined as (1-entropy) to be consistent with other scores. It reports the average level of uncertainty (or “information” or “surprise”) inherent in the possible outcomes of the variable and thereby quantifies amino acid diversity at a given position.

Stereochemically Sensitive Entropy was calculated as for Shannon Entropy except that amino acids were grouped based on the rules (['V','L', 'I','M'], ['F','W','Y'], ['S','T'], ['N','Q'], ['H','K','R'], ['D','E'], ['A','G'], ['P'], ['C']), as described previously (34). Amino acids within the same group are treated as a single entity. Variation is only considered when an amino acid from one of these groups is replaced with an amino acid from another group. Therefore, Stereochemically Sensitive Entropy quantifies physiochemical similarity rather than chemical identity.

JSD was calculated as first applied in (36) and as summarized as Capra07 (see Table 4 in (99)). JSD quantifies the similarity between two probability distributions. In CoSMoS.c., we used BLOSUM62 (BLOcks SUbstitution Matrix62) as the background amino acid distribution, which approximates the distribution of amino acid sites subject to no evolutionary pressure. This matrix is built using sequences with less than 62% similarity (sequences with ≥62% identity are clustered). BLOSUM62 is the default matrix for protein BLAST. This is also the designated background distribution in Capra07 and was shown to have broad applicability (36). Therefore, JSD reports how much we expect the amino acid sequence to change assuming no evolutionary constraint. In other words, JSD emphasizes selection pressure rather than chemical similarity. If the observed changes differ substantially from expectation (BLOSUM62), this suggests the presence of selection pressure and functional importance. This might also arise from distinct amino acid propensities when comparing ordered protein regions, from which the BLOSUM62 matrices were constructed, and disordered regions, where most modifications are likely to occur. This is unlikely to impact our results, as we are comparing structurally similar paralogous proteins. In addition, we are using multiple score algorithms to support our conclusions.

PhyloZOOM was calculated using the ‘zoom’ method, as described in (35). It is based on Shannon Entropy and uses a prebuilt phylogenetic tree based on the biallelic SNPs of the 1012 strains (32). This algorithm integrates strain phylogeny information into the Shannon Entropy conservation score calculation and in this way, corrects for relatedness among strains. It imposes a high penalty if a mutation occurs in a comparison strain closely related to the reference strain (in our case, S288C) and imposes a low penalty if it occurs in a distantly related comparison strain. Therefore, PhyloZOOM weights evolutionary relatedness on top of chemical identity.

Karlin Substitution Matrix was calculated with Karlin Normalization as described in (37). The range of the score is (−1,1). This was then reranged to (0,1) to be consistent with other scores. The Karlin Substitution Matrix algorithm emphasizes the probability of amino acid substitutions. Karlin Substitution Matrix sums the weights set for each possible substitution pair based on a background distribution, which sets the rules for how a substitution is penalized. CoSMoS.c. uses BLOSUM62 to be consistent with protein BLAST, wherein a rare substitution is penalized more than a common substitution. However, the background distribution can be substituted, for example with PAM30 (100), if another set of penalty rules is preferred. Therefore, Karlin Substitution Matrix quantifies the likeliness of observed substitutions, rather than quantifying chemical or biological properties of a given amino acid.

Thus, all scores were normalized to (0,1) with 0 being random and 1 being perfectly conserved. If gap penalty was applied, Conservation Score was calculated as Score∗(nongap amino acids percentage). Gaps were defined as noncanonical amino acids X,B,Z or “–“ produced from multisequence alignment. Gap penalty is inherent in the Karlin Substitution Matrix algorithm; therefore, no additional gap penalty was applied.

### Statistical tests for sequence conservation scores

We performed two different statistical tests because the underlying distribution has a pairing structure for each modified target protein and its unmodified paralog. One possibility is that the target protein score distribution is much larger than that of its paralog, and the distributions do not overlap (Fig. S7*A*). In this situation, the pairing structure does not matter and the target protein score is unambiguously larger than that of its paralog (Fig. S7*D*). In this instance, we applied a one-sided, paired Mann-Whitney-Wilcoxon Test (101), which determines whether the target protein conservation score distribution is significantly larger than the unmodified paralog conservation score distribution, without assuming that they follow a normal distribution. We used the paired test because the comparison is between the means of paired observations that have a relationship between the two groups (modified target and unmodified paralogs). Hereafter, we refer to this as distribution mean test.

A second possibility is that modified target proteins and unmodified paralogs have conservation score distributions that overlap substantially (Fig. S7*B*), but with a pairing structure such that the target protein score is usually higher than that of its paralog (advantage) (Figs. 4, *A* and *B* and S7*D*). Another possibility is that the pairing structure could disadvantage the target protein, such that the target protein score is usually lower than that of its paralog (Fig. 4, *C* and *D*). To test whether the pairing structure matters, we applied the Monte Carlo Simulation. We first calculated the percentage of pairings for which the modified target protein scores were greater than that of their unmodified paralogs, using established paralog gene pairs (“authentic pairs”). We then shuffled the pairings of target proteins and paralogs and calculated the percentage as before and repeated this 10,000 times. Lastly, we calculated the frequency for which “authentic pairs” was greater than that of the simulations. Therefore, the values are precise to 0.0001. This method allowed us to determine if the authentic pairing structure confers an advantage for the target proteins. Hereafter, we refer to this as “paralog pairing test”.

A third possibility is that a modified target protein and unmodified paralog have conservation score distributions that overlap partially (Fig. S7*C*). In this case, the distribution mean test will reveal whether the mean difference is statistically significant. Under these same circumstances, the results of the paralog pairing test might not be significant, which would indicate that the pairing structure is not contributing to the advantage of the target protein (Fig. S7*D*).

If the mean conservation score distribution of modified target proteins is substantially larger than that of the unmodified paralogs, such that the distributions do not overlap, the result of the distribution mean test would undoubtedly be statistically significant. In this situation, the result of the paralog pairing test will not be significant, as no matter how the pairing is structured, all target proteins will have a higher mean conservation score than that of the paralogs. Therefore, in this scenario, the target protein has a significantly higher mean conservation score than its paralog, but the pairing structure does not contribute to the significance of that difference.

If the mean conservation score distribution of the modified target proteins is larger but overlapping with that of the unmodified paralogs, the results of both the distribution mean test and the paralog pairing test could be statistically significant. In this situation, while the mean distribution of target proteins is significantly larger than that of the unmodified paralogs, the pairing structure could also contribute to the significance of the difference. If the mean conservation score distribution of the modified target protein is substantially overlapping with that of the unmodified paralog, the results of the paralog pairing test could still be statistically significant, while the results of the distribution mean test will undoubtedly not be significant. Although the distributions are similar, the pairing structure can still result in the modified target protein having a significantly larger mean conservation score than that of the unmodified paralog (See Figs. 4 and S7).

### Analysis of sequence motifs near sites of posttranslational modification

We made two comparisons, (1) whether there are significant differences in amino acid distribution, for a specific site, between target and paralog and (2) whether the target or paralog distributions are significantly different from the background frequencies based on BLOSUM62. Amino acid frequencies for each of the four positions upstream and downstream of each target modification site and the corresponding regions of the unmodified paralog protein were counted. The Chi-square test (chisq.test in stats package of R, with arguments: simulate.p.value = T, B = 10,000) was used to determine whether the categorized distributions, that is, amino acid counts, were significantly different comparing (1) target proteins and paralogs and (2) either target proteins or paralogs with BLOSUM62. To further determine which category (amino acid) contributes to the significant differences if any, post hoc analysis was performed using the standardized residuals (stdres from chisq.test results, using pnorm to get the cumulative probability at the value of stdres, then using Benjamini-Hochberg for multicomparison correction).

### Analysis of posttranslation modifications

Modifications in the proteome of *S. cerevisiae* were obtained from annotated lists in the SGD database (https://yeastmine.yeastgenome.org/yeastmine/begin.do) on September 27, 2021 and were assigned to 550 paralog sequences also obtained from SGD YeastMine on July 14, 2020.

### Analysis of structural motifs near sites of posttranslational modification

AlphaFold protein structure prediction for *S. cerevisiae* was downloaded from AlphaFold website (https://alphafold.ebi.ac.uk/download) (53, 54). The uncompressed.pdb files generated by AlphaFold were used as input data for protein secondary structure assignment with STRIDE as a stand-alone program (July 7th, 2022) (http://webclu.bio.wzw.tum.de/stride/) (55). STRIDE assignments of secondary structure and residue solvent accessible area were extracted from “Detailed secondary structure assignment section”. These secondary structure predictions were then matched to each of the modified target and unmodified paralog pairs. Secondary structure length is defined as the number of amino acids with an uninterrupted stretch of secondary structure, for a given site of modification or for the corresponding site of the unmodified paralog. For each modified target and unmodified paralog pair, the difference between the residue solvent accessible area and secondary structure length was calculated and presented as a boxplot for each set of target and paralog secondary structures (*e.g.*, turn-turn, turn-coil, etc.).

### Analysis of kinase–substrate interactions

Kinase and substrate interaction data were downloaded from the Yeast KID-kinase interaction database (http://www.moseslab.csb.utoronto.ca/KID/index.php) on May 27, 2022 (56). A total of 31,155 interactions were downloaded for all substrates and including all types of experimental evidence. The dataset included 127 kinases in total. For each of the 550 paralog pairs, the number of kinases that interacted with both proteins (double interaction) and the number that interacted with only one of the two proteins (single interaction) were counted. The single interaction ratio was then defined as (number of kinases with single interaction)/(number of kinases with single interaction + number of kinases with double interaction).

## Data availability

This manuscript uses data from public repositories. All original data are contained within the manuscript. CoSMoS.c. code has been deposited with GitHub: https://github.com/Shuang-Plum/YeastMotifConserv.

## Supporting information

This article contains supporting information.

## Conflict of interest

The authors declare that they have no conflicts of interest with the contents of the article.
